# The color masking ability of a zirconia ceramic on the substrates with different values

**DOI:** 10.15171/joddd.2017.002

**Published:** 2017-03-15

**Authors:** Farhad Tabatabaian, Mahdiye Javadi Sharif, Farhood Massoumi, Mahshid Namdari

**Affiliations:** ^1^Assistant Professor, Department of Prosthodontics, School of Dentistry, Shahid Beheshti University of Medical Sciences, Tehran, Iran; ^2^Postgraduate Student, School of Dentistry, Shahid Beheshti University of Medical Sciences, Tehran, Iran; ^3^Assistant Professor, Department of Prosthodontics, School of Dentistry, Shahid Beheshti University of Medical Sciences, Tehran, Iran; ^4^Assistant Professor of Biostatistics, Department of Community Oral Health, School of Dentistry, Shahid Beheshti University of Medical Sciences, Tehran, Iran

**Keywords:** Color, spectrophotometry, visual perception, Y-TZP ceramic

## Abstract

***Background.*** The color masking ability of a restoration plays a significant role in coveringa discolored substructure; however, this optical property of zirconia ceramics has not been clearly determined yet. The aim of this in vitro study was to evaluate the color masking ability of a zirconia ceramic on substrates with different values.

***Methods.*** Ten zirconia disk specimens,0.5 mm in thickness and 10 mm in diameter, were fabricated by a CAD/CAM system. Four substrates with different values were prepared, including: white (control), light grey, dark grey, and black. The disk specimens were placed over the substratesfor spectrophotometric measurements. A spectrophotometer measured the L^*^, a^*^, and b^*^ color attributes of the specimens. Additionally, ΔE values were calculated to determine the color differences between each group and the control,and were then compared with the perceptional threshold of ΔE=2.6. Repeated-measures ANOVA, Bonferroni, and one-sample t-test were used to analyze data. All the tests were carried out at 0.05 level of significance.

***Results.*** The means and standard deviations of ΔE values for the three groups of light grey, dark grey and black were 9.94±2.11, 10.40±2.09, and 13.34±1.77 units, respectively.Significant differences were detected between the groups in the ΔE values (P<0.0001).The ΔE values in all the groups were more than the predetermined perceptional threshold(ΔE>2.6) (P<0.0001).

***Conclusion.*** Within the limitations of this study, it was concluded that the tested zirconia ceramic did not exhibit sufficient color masking ability to hide the grey and black substrates.

## Introduction

Metal ceramic restorations have shown long-term success due to good mechanical properties.^1,2^However, achieving a natural appearance is more challenging with a metal-ceramic restoration than an all-ceramic restoration due tothe fact that metal copings prevent light transmission.^3,4^ This has led to an increase in the use of non-metallic restorations such as zirconia-based restorations.^5^ Zirconiacrowns combine the benefits of metal restorations, such as minimally invasive tooth preparation and simple cementation, with those of all-ceramic crowns, such as low thermal conductivity and adequate translucency.^6,7^ However, the high translucency of a ceramic is not necessarily a benefit,especiallyin cases with discolored teeth, metallic core materials, colored dental substrates,^8^and even titanium abutments.^9^ In these cases a ceramic with color masking ability of its background should be reasonably applied to obtain acceptable esthetic outcomes. The masking ability has been defined as the ability to hide a colored background.^5^

A method to evaluate the masking ability of restorative materials is to measure a ΔE color difference in CIELab color system. In this color system the color attributes of L^*^, a^*^, and b^*^ define lightness, red/green value, and yellow/blue value, respectively, which can be measured via spectrophotometry. The ΔE is calculated to determine color differences using this formula:

ΔE^*^_ab_=[(L^*^_2_-L^*^_1_)^2^+(a^*^_2_-a^*^_1_)^2^+(b^*^_2_-b^*^_1_)^2^]^1/2^

which is the most commonly used formula forΔE.^5^ This formula can detect even a small amount of color difference between natural dentition and restorations.^10^ An opaquematerial with ideal masking ability has a ΔE color difference close to zero when being placed over a black and white substrate.^8^ In this case the color of the material is not affected by the substrate’s color. As spectrophotometers and colorimeters can recognize even a small amount of color change which cannot be detected by human eyes, limits have been defined for the perceptional threshold and the acceptable clinical threshold based on the ΔE value.^5^ It has been considered that the acceptable clinical threshold is more than the perceptional threshold.^11^ If the ΔE color change is more than the threshold, a color match will be rejected. The perceptional threshold range is1‒5.5of ΔE in the literature.^12-14^

Several studies have evaluated the optical properties of different ceramic materials.^15-18^Some investigations have assessed various factors affecting the final color of zirconia restorations, including substrates,^19-22^ cements,^21,23^ ceramic veneers and their thicknesses,^24-26^ glazing process,^24^and laboratory techniques.^27^

Suputtamongkol et al^19^concluded that the color of a substructure affected the color of posterior zirconia-based restorations on a metal post and core or a prefabricated post and acomposite resin core, ranging from 1.2 to 3.1 of ΔE. Choi & Razzoog^20^evaluated the masking ability of zirconia ceramics with and without porcelain veneer and concluded that the unveneered zirconia ceramic was rather capable of masking the different tested substrates. Oh & Kim^22^ concluded that abutment shade, ceramic thickness and coping type affected the color resulting from zirconia restorations.Pecho et al^28^ concluded that clinicians shouldpay attention tothe optical property differences betweenzirconiaceramics and human dentin to achieve optimal esthetics in restorative dentistry. Kurtulmus-Yilmaz and Ulusoy^29^showed that zirconia ceramics were less translucent than lithium disilicate glass ceramic, and had partial translucency with some translucency differences among the zirconia systems. Tuncel et al^30^reported that the translucency of zirconia was influenced by the coloring procedure and the grain size. The masking ability of a zirconia ceramic is related to its color coverage over underlying structures, including cement and dental substrates. Masking a dental substrate with cements may not be feasible because different shades do not exist for all the cements; in addition, availability of different cement shades allows minor esthetic corrections.^21^Therefore the color masking ability of a zirconia ceramic on different substrates may be essential to achieve proper clinical results. The L^*^attribute, which is related to the value or lightness of a ceramic or a substrate, is the most effective factor in the resultant color.^20^ Therefore, the aim of this in vitro study was to evaluate the color masking ability of a zirconia ceramic on the substrates with different values. The null hypothesis was that the examined zirconia ceramic would show sufficient color masking ability to hide the tested substrates.

## Methods

A sample of maximum 10 is needed in a repeated-measure design with 3 replicates and the effect size of 0.81 (variance within groups=4 and variance explained by the effect=2.6), with α=0.05 and β=0.1. A G power of 3.1.3 was implemented for sample size calculation.

Therefore, 10 zirconia disk specimens were fabricated. The disk specimens were placed onto four substrates with different values, including: white (W), light grey (LG), dark grey (DG) and black (B). Spectrophotometric measurements were performed on the specimens.

A CAD/CAMsystem (CORITEC 250i, imes-icore GmbH, Eiterfeld, Germany) milled zirconia blocks (Luminesse High Strength 98mm Discs #5113, Talladium, Valencia, CA, USA) to prepare zirconia disks. The disks were 0.5 mm in thickness and 10 mm in diameter.^22^All the zirconia disks were sintered at 1500°C for a 12-hour process in a sintering furnace (iSINT HT, Imes-Icore GmbH, Eiterfeld, Germany). A micrometer (293 MDC-MX Lite, Mitutoyo Corporation, Tokyo, Japan) with an accuracy of 0.002 mm was employed to measure the thicknesses of the disks. The disks were adjusted to a thickness of 0.5±0.01 mm. An adjustment and polishing kit (BruxZir, Glidewell Direct, Irvine, CA, USA) was used to reduce the thicknesses according to the above-mentioned acceptable range. In case of lack of the acceptable thickness, the disk was excluded from the study. The zirconia disks were polished, cleaned in an ultrasonic bath (Elmasonic S-30, Dentec, North Shore, Australia) containing 98% ethanol for 15 minutes and finally dried.

**Figure 1.**
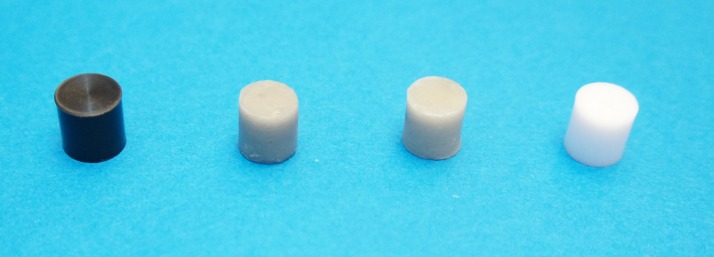
The tested substrates.

Four cylindrical substrates with different values, including white (W), light grey (LG), dark grey (DG) and black (B), were prepared. The substrates were 10 mm in diameter and 10 mm in height.^22^ White and black Teflon materials(PTFE, Omnia Plastica SPA, Busto Arsizio, Italy) were milled to fabricate the white and black substrates according to the above-mentioned dimensions. In order to fabricate grey substrates, a putty silicone impression (Speedex, Coltene, Altstatten, Switzerland) was taken from the black substrate to prepare a mold. Half a gram and one gram of carbon graphite were separately added to the liquid of an auto-polymerized acrylic resin (Alike, GC Corporation, Tokyo, Japan), to make the light and dark grey substrates, respectively.^20^ The liquid and powder of the acrylic resin were mixed and applied to the mold. After 5 minutes the polymerization of the acrylic resin was completed. The grey acrylic substrates were polished with 800-grit silicon carbide abrasive papers for 10 minutes. All the substrates were cleaned in the same ultrasonic bath containing 98% ethanol for 15 minutes (Figure 1). The CIELab values of the substrates were measured (W: L^*^=92.1, a^*^=-1.7, b^*^=0.7; LG; L^*^=57.6, a^*^=-0.6, b^*^=4.3; DG: L^*^=39.8, a^*^=-0.5, b^*^=2.5; B:L^*^=9.4, a^*^=-0.9, b^*^=-1.1)

A spectrophotometer (Spectro Shade Micro, MHT, Verona, Italy) was employed for spectrometric measurements.^31^ A putty silicone material (Speedex, Coltene, Altstatten, Switzerland) was adapted to the mouthpiece of the spectrophotometer to match the conditions of spectrophotometry for all the specimens and to prevent external light. The specimens were located at the center of this putty mold. Before each measurement the spectrophotometer was calibrated by the white and green calibration plates, respectively. The disks were placed on the substrates with a water drop in between to prevent refraction of light.^5^ Each disk specimen was placed on each of the four substrates and the color measurements were carried out. All the color measurements were conducted at the center of the specimens marked by a pen on the monitor screen of spectrophotometer, and the color attributes of L^*^, a^*^, and b^*^ were recorded for each specimen. ΔE was calculated to determine the color differences of a disc on different substrates. To compare the three substrates of LG, DG and B with the substrate W, the ΔE values were measured in three situations, including W-LG, W-DG and W-B. The aforementioned formula was employed to calculate ΔE. The ΔE=2.6for perceptional threshold was hypothesized in this study.^13,14^

A normal distribution of data was confirmed in all the groups by Kolmogorov-Smirnov test (P>0.05). SPSS 21 (SPSS Inc., Chicago, IL, USA) was used for the analysis of data. In order to compare the color attributes of L^*^, a^*^, b^*^ and ΔE in the four groups of W, LG, DG and B, repeated-measures ANOVA was employed. Pairwise comparisons of the groups were performed by Bonferroni test. STATA (StataCorp LP, Lake way, TX, USA) was used to compare the ΔE values with the threshold of 2.6 using one-sample t-test. All the tests were carried out at 0.05 level of significance.

## Results

**Figure 2.**
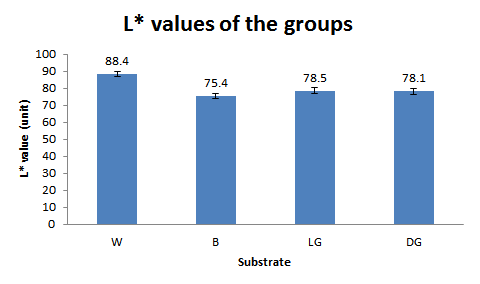
The means and standard deviations of the L^*^ values in the four groups.

The means and standard deviations of the L^*^ values for the four groups of W, LG, DG, and B were 88.35±1.46, 78.46±1.71, 78.06±1.66, and 75.38±1.35units, respectively (Table 1) (Figure 2). Repeated-measures ANOVA detected significant differences between the groups (P<0.0001). Pairwise comparisons of the four groups revealed significant differences between all the groups (P<0.0001), except between LG and DG (P=0.652).

**Table 1:** Measures of the color attributesof specimens in the four groups

**Substrate**	**Color attributes**	**Mean**	**Standard deviation**	**Minimum**	**Maximum**	**%95 Confidence Interval**
	L^*^	88.35	1.46	85.40	90.50	(87.30,89.39)
**White** **(W)**	a^*^	-0.40	0.42	-1.10	0.30	(-0.69,-0.10)
	b^*^	3.38	0.36	2.90	4.10	(3.11,3.64)
	L^*^	75.38	1.35	73.20	77.10	(74.41,76.34)
**Black** **(B)**	a^*^	-0.36	0.45	-0.80	0.80	(-0.68,-0.03)
	b^*^	0.40	0.49	-0.30	1.40	(0.05,0.75)
	ΔE	13.34	1.77	9.64	15.94	(12.06,14.60)
	L^*^	78.46	1.71	76.00	80.20	(77.24,79.68)
**Light Grey** **(LG)**	a^*^	-0.32	0.48	-0.80	0.90	(-0.66,0.027)
	b^*^	2.61	0.41	2.20	3.40	(2.31,2.90)
	ΔE	9.94	2.11	5.88	12.80	(8.43,11.45)
	L^*^	78.06	1.66	75.20	79.60	(76.87,79.24)
**Dark Grey ** **(DG)**	a^*^	-0.28	0.52	-0.80	1.00	(-0.65,0.09)
	b^*^	2.06	0.44	1.50	2.70	(1.74,2.37)
	ΔE	10.40	2.09	5.98	13.86	(8.89,11.89)

**Figure 3.**
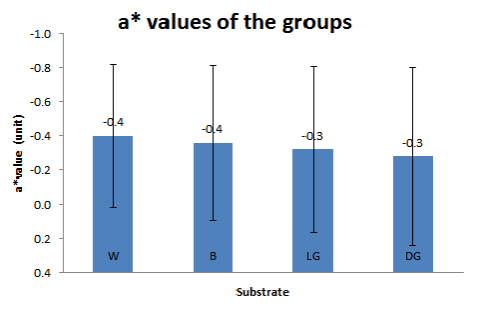
The means and standard deviations of the a^*^ values in the four groups.

The means and standard deviations of a^*^ values for the four groups of W, LG, DG and B were-0.40±0.42, -0.32±0.48, -0.28±0.52, and-0.36±0.45units, respectively (Table 1) (Figure 3). Repeated-measures ANOVA detected no significant differences between the groups (P=1).

**Figure 4.**
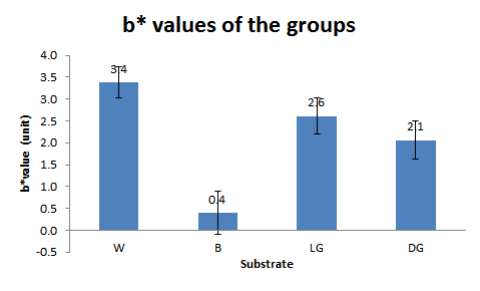
The means and standard deviations of the b^*^ values in the four groups.

The means and standard deviations of b^*^values for the four groups of W, LG, DG and B were 3.38±0.36, 2.61±0.41, 2.06±0.44, and 0.40±0.49units, respectively (Table 1) (Figure 4). Repeated-measures ANOVA detected significant differences between the groups (P<0.0001). Pair wise comparisons of the four groups showed significant differences between all the groups (P<0.0001), except between LG and W (P=0.09).

**Figure 5.**
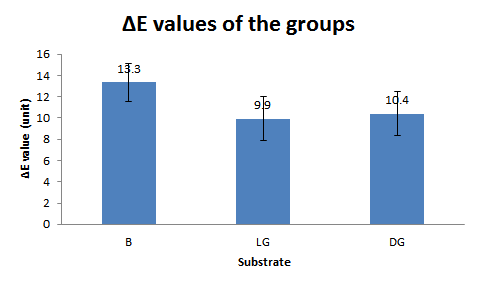
The means and standard deviations of the ΔE values.

The means and standard deviations of the ΔE_W-LG_, ΔE_W-DG_, and ΔE_W-B_ values were 9.94±2.11, 10.40±2.09, and 13.34±1.77units, respectively (Table 1) (Figure 5). Repeated-measures ANOVA showed significant differences between the groups (P<0.0001). Pair wise comparisons of the groups revealed significant differences between all the groups (P<0.0001), except between LG and DG (P=0.469). In order to compare the means of the ΔE_W-LG_, ΔE_W-DG_, and ΔE_W-B_ values with the threshold of ΔE=2.6, one-sample t-test (one-sided) was employed. The null hypothesis of µ≤2.6 was rejected for ΔE_W-LG_, ΔE_W-DG_ and ΔE_W-B_ (P<0.0001).

## Discussion

Based on the results of this study, the tested zirconia ceramic upon the tested substrates demonstrated perceptible color differences and did not show sufficient color masking ability to hide the grey and black substrates. Hence, the null hypothesis of the study was refuted.

The L^*^ values decreased in all the groups compared with the W. This decrease was the highest in the B, while there was no significant difference between the LG and the DG in this respect. As the L^*^ value expresses the lightness of an object, a black substrate reasonably causes the highest decrease in this color attribute. This is due to optical properties of zirconia ceramic which allows for light transmission.

The a^*^values increased in all the groups compared with the W, though this increase was not statistically significant. This shows that grey and black substrates do not affect the a^*^color attribute. As the a^*^ color attribute defines red/green value, the result seems rational.

The b^*^values decreased in all the groups compared with the W. The decrease of the b^*^ value was the highest in the B. This may be due to the natural b^*^ value of a black substrate and its effect on the zirconia ceramic.

All the ΔE values (ΔE_W-LG_, ΔE_W-DG_ and ΔE_W-B_) were more than the threshold of ΔE=2.6. This demonstrated that the color changes induced by the substrates were beyond the perceptional threshold. Tracing the L^*^, a^*^, b^*^ and ΔE values (Table 1) indicated that the highest amount of difference was related to the L^*^ attribute. Accordingly the substrate types had the highest impact on the L^*^ attribute. Therefore the ΔE changes had mainly been derived from this attribute (Table 1).

Suputtamongkol et al^19^ reported that the color of a background substructure could affect the color of zirconia-based restorations on a metal post and core or a prefabricated post and composite resin core. Moreover minor color changes of zirconia crowns were disclosed by measuring ΔE.^19^Despite some differences between the above-mentioned study and the present investigation like ceramic brand, ceramic thickness, layered zirconia versus zirconia coping, thresholds and substrate types, both investigations revealed that the zirconia ceramic was rather capable of masking its substrate.

Oh & Kim^22^ assessed the effects of abutment shade, ceramic thickness, and coping type on the color of zirconia-based restorations. The abutments were prepared with gold alloy, nickel-chromium alloy, and composite resins with different shades. The mean ΔE value of Lava zirconia between the A2 composite resin and gold alloy abutments was higher than those between the A2 composite resin and other abutments (ΔE =5.5). It was concluded that the color of the tested zirconia ceramic was affected by its substrate. A comparable consequence was gained from the present study, though the substrates and zirconia ceramics used were not similar.

Choi & Razzoog^20^assessed the masking ability of a zirconia ceramic with and without porcelain veneer. The color differences induced by zirconia ceramic and porcelain veneer were compared with the substrates alone, and it was concluded that the unveneered zirconia ceramic was rather capable to mask the different tested substrates. However, in the present study the color differences induced by the substrates were compared with a white substrate and the results revealed that the tested zirconia ceramic could not hide the substrates. In other words, Choi &Razzoog^20^ calculated the ΔE between the substrate (as a control) and the zirconia ceramic with the substrate, while we calculated the ΔE between the zirconia ceramic with the white substrate (as a control) and the zirconia ceramic with the other substrates. The difference in the results might be attributed to the mentioned methodological approaches. It should be considered that incomplete masking ability of a ceramic on discolored background substructures might lead to unpleasant esthetic results. On the other hand, the highest color changes, reported by Choi & Razzoog,^20^ occurred in the L^*^ and b^*^ values. Similarly the highest amount of color differences were caused by the L^*^ attribute in the current investigation.

Although Kurtulmus-Yilmaz and Ulusoy^29^showed that zirconia-based all-ceramic systems had lower translucency than lithium disilicate-based glass ceramics, this lower level of translucency of zirconia is not sufficient to hide the substrate color according to the results of this study. Tuncel et al^30^showed that zirconia as a framework material had smaller grain size and more translucency compared to monolithic zirconia. In addition, colored zirconia framework material exhibited similar grain size and was less translucent than non-colored zirconia framework material.^30^ The results of the present study, which showed the effect of substrate color on the zirconia ceramic color, were consistent with those of a study by Tuncel et al,^30^ indicating the high translucency of non-colored zirconia framework.

Based on the results of this study, the grey and black substrates can change the color of zirconia core beyond the perceptional threshold. Thus, zirconia-based restorations may be contraindicated on the black and grey discolored teeth, or the negative effects of these discolorations might be reduced by increasing the zirconia core thickness, applying sufficient porcelain veneers, and using suitable luting agents.

Light transmission via a zirconia core structure can be predictable in zirconia-based restorations, and there forecements and dental substrates might affect the color of these restorations.^32^ The present study assessed the specific effects of three substrates with different values in this respect. In addition, the zirconia core veneering materials may affect the color of zirconia-based restorations, which were not evaluated in this study. Therefore consideration of this factor is suggestedin future studies. This research had some limitations such as testing a non-coloredzirconia ceramic without veneer. More investigations are suggested in this respect.

## Conclusion

Within the limitations of this study it was concluded that the tested zirconia ceramic did not exhibit sufficient color masking ability to hide the grey and black substrates.

## Acknowledgments

This paper has been entirely drawn up from a “M.D. Thesis” which was successfully completed by Dr. Mahdiye Javadi Sharif under the supervision of Dr. Farhad Tabatabaian.

## Authors’ contributions

FT contributed to the concept and design of the work. The acquisition, analysis, and interpretation of data were accomplished by FT, MJS, and MN. FT,FM and MJSdrafted and revised it critically for intellectual content. All the authors read and approved the final manuscript.

## Funding

This project was supported and funded by the authors.

## Competing interests

The authors declare no competing interests with regards to the authorship and/or publication of this article.

## Ethics approval

Not applicable.
